# 1-(4-Fluoro­phen­yl)-2-(phenyl­sulfon­yl)ethanone

**DOI:** 10.1107/S1600536812019666

**Published:** 2012-05-05

**Authors:** Hatem A. Abdel-Aziz, Hazem A. Ghabbour, Suchada Chantrapromma, Hoong-Kun Fun

**Affiliations:** a Department of Pharmaceutical Chemistry, College of Pharmacy, King Saud University, PO Box 2457, Riyadh 11451, Saudi Arabia; b Crystal Materials Research Unit, Department of Chemistry, Faculty of Science, Prince of Songkla University, Hat-Yai, Songkhla 90112, Thailand; c X-ray Crystallography Unit, School of Physics, Universiti Sains Malaysia, 11800 USM, Penang, Malaysia

## Abstract

In the title compound, C_14_H_11_FO_3_S, the unit comprising the ethanone and 4-fluoro­phenyl groups is essentially planar, with an r.m.s. deviation of 0.0084 (2) Å for the ten non-H atoms, and it makes a dihedral angle of 37.31 (10)° with the phenyl ring. In the crystal, mol­ecules are linked by pairs of weak C—H⋯O hydrogen bonds into inversion dimers with *R*_2_^2^(16) graph-set motifs. The dimers are stacked along the *b* axis through further C—H⋯O hydrogen bonds.

## Related literature

For bond-length data, see: Allen *et al.* (1987 ▶). For background to the chemistry of aryl­sulphones, see: Abdel-Aziz *et al.* (2009 ▶, 2010 ▶); Grandison *et al.* (2002 ▶); Silvestri *et al.* (2000 ▶); Stephens *et al.* (2001 ▶); Xiang *et al.* (2007 ▶). For related structures, see: Abdel-Aziz *et al.* (2011 ▶, 2012 ▶). For hydrogen-bond motifs, see: Bernstein *et al*. (1995 ▶).
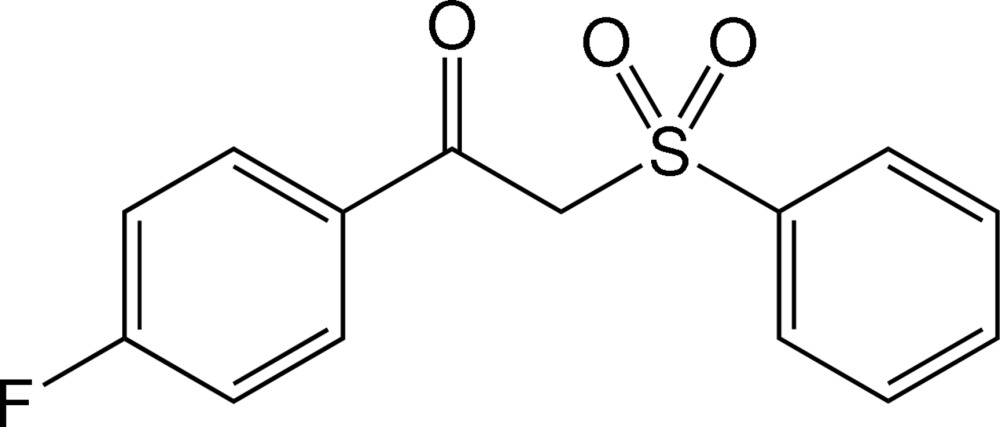


## Experimental

### 

#### Crystal data

C_14_H_11_FO_3_S
*M* *_r_* = 278.30
Monoclinic, 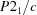

*a* = 13.7046 (3) Å
*b* = 5.3216 (1) Å
*c* = 20.4401 (5) Å
β = 119.612 (2)°
*V* = 1296.01 (6) Å^3^
*Z* = 4
Cu *K*α radiation
μ = 2.36 mm^−1^
*T* = 296 K
0.58 × 0.23 × 0.07 mm

#### Data collection

Bruker SMART APEXII CCD area-detector diffractometer
Absorption correction: multi-scan (*SADABS*; Bruker, 2009 ▶) *T*_min_ = 0.341, *T*_max_ = 0.852
8880 measured reflections
2393 independent reflections
2090 reflections with *I* > 2σ(*I*)
*R*_int_ = 0.033

#### Refinement

*R*[*F* ^2^ > 2σ(*F* ^2^)] = 0.037
*wR*(*F* ^2^) = 0.105
*S* = 1.07
2393 reflections
173 parameters
H-atom parameters constrained
Δρ_max_ = 0.22 e Å^−3^
Δρ_min_ = −0.30 e Å^−3^

### 

Data collection: *APEX2* (Bruker, 2009 ▶); cell refinement: *SAINT* (Bruker, 2009 ▶); data reduction: *SAINT*; program(s) used to solve structure: *SHELXTL* (Sheldrick, 2008 ▶); program(s) used to refine structure: *SHELXTL*; molecular graphics: *SHELXTL*; software used to prepare material for publication: *SHELXTL* and *PLATON* (Spek, 2009 ▶).

## Supplementary Material

Crystal structure: contains datablock(s) global, I. DOI: 10.1107/S1600536812019666/is5133sup1.cif

Structure factors: contains datablock(s) I. DOI: 10.1107/S1600536812019666/is5133Isup2.hkl

Supplementary material file. DOI: 10.1107/S1600536812019666/is5133Isup3.cml

Additional supplementary materials:  crystallographic information; 3D view; checkCIF report

## Figures and Tables

**Table 1:** Hydrogen-bond geometry (Å, °)

*D*—H⋯*A*	*D*—H	H⋯*A*	*D*⋯*A*	*D*—H⋯*A*
C1—H1*B*⋯O3^i^	0.97	2.23	3.183 (2)	168
C5—H5*A*⋯O2^ii^	0.93	2.57	3.218 (3)	127

Symmetry codes: (i) 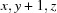; (ii) 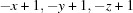.

**Fig. 1.**
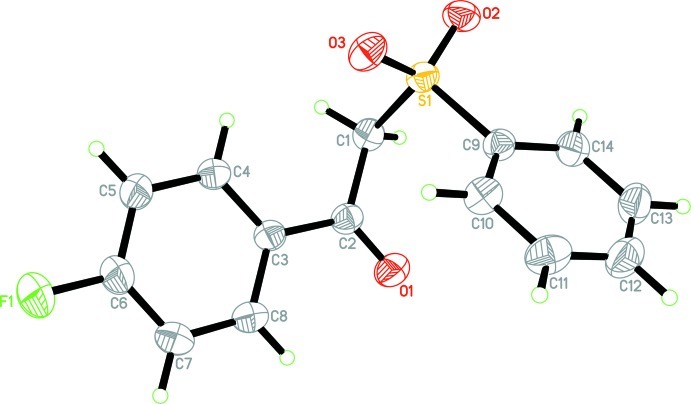
The molecular structure of the title compound, showing 30% probability displacement ellipsoids and the atom-numbering scheme.

**Fig. 2.**
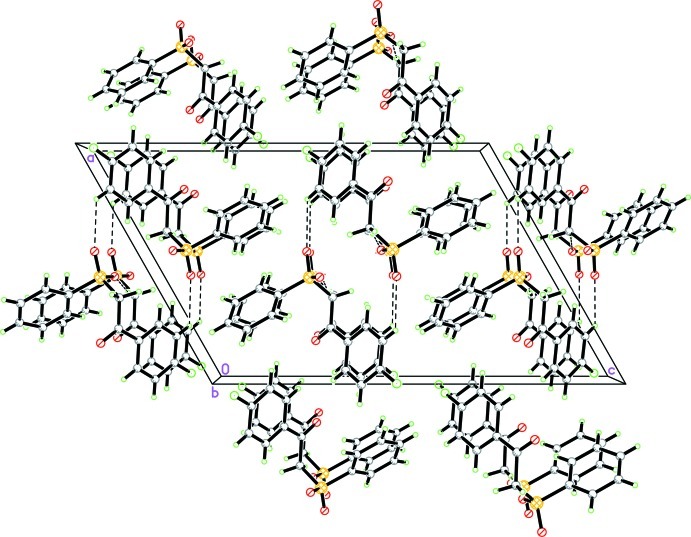
A crystal packing diagram of the title compound viewed along the b axis, showing molecular layers parallel to the ac plane. The C—H···O hydrogen bonds are shown by dashed lines.

## supplementary crystallographic information

### Comment

Arylsulphones are an interesting class of non-nucleoside antiviral agents. A large number of them have been also shown various interesting biological activities (Abdel-Aziz *et al.*, 2009, 2010; Silvestri *et al.*, 2000; Stephens *et al.*, 2001) and found application in membrane technology for nanofiltration (Grandison *et al.*, 2002). During the course of our research on the medicinal chemistry of arylsulphones, the title compound(I) was synthesized and studied for its biological activity. Herein the crystal structure of (I) was reported.

The title molecule has a fold structure as indicated by the dihedral angle between the 4-fluorophenyl and phenyl rings being 36.98 (12)° (Fig. 1). The ethanone unit [C1/C2/O1] lies on the same plane with the 4-fluorophenyl ring with an *r.m.s*. deviation of 0.0084 (2) Å for the ten non-H atoms (C1–C8/O1/F1) and the dihedral angle between the ethanone plane and the 4-fluorophenyl ring being 1.2 (2)°. The environment of S atom is a distorted tetrahedral geometry [angles around S atom are 105.67 (8)–118.24 (9)°] being surrounded by two O atoms, one C atom of the ethanone unit and one C atom of the benzene ring. The bond distances in (I) are within normal ranges (Allen *et al.*, 1987) and comparable to the related structures (Abdel-Aziz *et al.*, 2011, 2012).

In the crystal packing (Fig. 2), the molecules are linked by pairs of weak C···H···O_sulfonyl_ interactions (Table 1) into inversion dimers with *R*_2_^2^(16) graph-set motifs (Bernstein *et al.*, 1995) and these dimers are arranged into layers parallel to the *ac* plane and stacked along the *b* axis.

### Experimental

The title compound was prepared according to the reported method (Xiang *et al.*, 2007). Colorless plate-shaped single crystals suitable for an *X*-ray structural analysis were obtained by slow evaporation from an ethanol solution at room temperature.

### Refinement

H atoms were placed in calculated positions with *d*(C—H) = 0.93 for aromatic and 0.97 Å for CH_2_ atoms. The *U*_iso_(H) values were constrained to be 1.2*U*_eq_ of the carrier atom.

### Crystal data

**Table:**

C_14_H_11_FO_3_S	*F*(000) = 576
*M**_r_* = 278.30	*D*_x_ = 1.426 Mg m^−^^3^
Monoclinic, *P*2_1_/*c*	Cu *K*α radiation, λ = 1.54178 Å
Hall symbol: -P 2ybc	Cell parameters from 2393 reflections
*a* = 13.7046 (3) Å	θ = 3.7–71.2°
*b* = 5.3216 (1) Å	µ = 2.36 mm^−^^1^
*c* = 20.4401 (5) Å	*T* = 296 K
β = 119.612 (2)°	Plate, colorless
*V* = 1296.01 (6) Å^3^	0.58 × 0.23 × 0.07 mm
*Z* = 4	

### Data collection

**Table:**

Bruker SMART APEXII CCD area-detector diffractometer	2393 independent reflections
Radiation source: fine-focus sealed tube	2090 reflections with *I* > 2σ(*I*)
Graphite monochromator	*R*_int_ = 0.033
φ and ω scans	θ_max_ = 71.2°, θ_min_ = 3.7°
Absorption correction: multi-scan (*SADABS*; Bruker, 2009)	*h* = −16→16
*T*_min_ = 0.341, *T*_max_ = 0.852	*k* = −5→6
8880 measured reflections	*l* = −24→25

### Refinement

**Table:**

Refinement on *F*^2^	Secondary atom site location: difference Fourier map
Least-squares matrix: full	Hydrogen site location: inferred from neighbouring sites
*R*[*F*^2^ > 2σ(*F*^2^)] = 0.037	H-atom parameters constrained
*wR*(*F*^2^) = 0.105	*w* = 1/[σ^2^(*F*_o_^2^) + (0.0503*P*)^2^ + 0.2892*P*] where *P* = (*F*_o_^2^ + 2*F*_c_^2^)/3
*S* = 1.07	(Δ/σ)_max_ = 0.001
2393 reflections	Δρ_max_ = 0.22 e Å^−^^3^
173 parameters	Δρ_min_ = −0.30 e Å^−^^3^
0 restraints	Extinction correction: *SHELXTL* (Sheldrick, 2008), Fc^*^=kFc[1+0.001xFc^2^λ^3^/sin(2θ)]^-1/4^
Primary atom site location: structure-invariant direct methods	Extinction coefficient: 0.0041 (5)

### Special details

**Table:**

Geometry. All esds (except the esd in the dihedral angle between two l.s. planes) are estimated using the full covariance matrix. The cell esds are taken into account individually in the estimation of esds in distances, angles and torsion angles; correlations between esds in cell parameters are only used when they are defined by crystal symmetry. An approximate (isotropic) treatment of cell esds is used for estimating esds involving l.s. planes.
Refinement. Refinement of F^2^ against ALL reflections. The weighted R-factor wR and goodness of fit S are based on F^2^, conventional R-factors R are based on F, with F set to zero for negative F^2^. The threshold expression of F^2^ > 2sigma(F^2^) is used only for calculating R-factors(gt) etc. and is not relevant to the choice of reflections for refinement. R-factors based on F^2^ are statistically about twice as large as those based on F, and R- factors based on ALL data will be even larger.

### Fractional atomic coordinates and isotropic or equivalent isotropic displacement parameters (Å^2^)

**Table:**

	*x*	*y*	*z*	*U*_iso_*/*U*_eq_	
S1	0.55567 (3)	0.70852 (8)	0.61500 (2)	0.04632 (18)	
F1	0.96706 (13)	0.1120 (4)	0.55007 (10)	0.1107 (6)	
O1	0.81880 (12)	0.9811 (3)	0.68962 (8)	0.0662 (4)	
O2	0.45075 (10)	0.8367 (3)	0.58825 (8)	0.0616 (4)	
O3	0.55394 (12)	0.4560 (2)	0.58997 (8)	0.0603 (4)	
C1	0.63784 (15)	0.8990 (3)	0.58896 (10)	0.0492 (4)	
H1A	0.6133	0.8700	0.5362	0.059*	
H1B	0.6236	1.0741	0.5944	0.059*	
C2	0.76418 (15)	0.8516 (4)	0.63472 (9)	0.0492 (4)	
C3	0.81547 (14)	0.6528 (4)	0.61084 (9)	0.0488 (4)	
C4	0.75317 (15)	0.5065 (4)	0.54707 (10)	0.0540 (4)	
H4A	0.6762	0.5324	0.5178	0.065*	
C5	0.80438 (17)	0.3233 (4)	0.52674 (11)	0.0630 (5)	
H5A	0.7629	0.2242	0.4844	0.076*	
C6	0.91764 (19)	0.2912 (5)	0.57045 (13)	0.0716 (6)	
C7	0.98184 (18)	0.4289 (6)	0.63456 (13)	0.0798 (7)	
H7A	1.0585	0.3991	0.6638	0.096*	
C8	0.93098 (16)	0.6100 (5)	0.65444 (12)	0.0660 (6)	
H8A	0.9735	0.7059	0.6974	0.079*	
C9	0.62748 (14)	0.7076 (3)	0.71439 (10)	0.0470 (4)	
C10	0.70719 (16)	0.5235 (4)	0.75248 (11)	0.0560 (4)	
H10A	0.7222	0.4007	0.7263	0.067*	
C11	0.76409 (19)	0.5262 (5)	0.83057 (12)	0.0691 (6)	
H11A	0.8182	0.4049	0.8573	0.083*	
C12	0.7406 (2)	0.7079 (5)	0.86849 (12)	0.0718 (6)	
H12A	0.7791	0.7089	0.9208	0.086*	
C13	0.6606 (2)	0.8885 (5)	0.82995 (13)	0.0733 (6)	
H13A	0.6452	1.0099	0.8563	0.088*	
C14	0.60266 (17)	0.8904 (4)	0.75166 (12)	0.0596 (5)	
H14A	0.5485	1.0119	0.7252	0.072*	

### Atomic displacement parameters (Å^2^)

**Table:**

	*U*^11^	*U*^22^	*U*^33^	*U*^12^	*U*^13^	*U*^23^
S1	0.0442 (3)	0.0421 (3)	0.0460 (3)	0.00262 (16)	0.01715 (19)	−0.00338 (15)
F1	0.0781 (9)	0.1412 (15)	0.1014 (11)	0.0380 (9)	0.0356 (8)	−0.0343 (10)
O1	0.0622 (8)	0.0691 (10)	0.0584 (8)	−0.0055 (7)	0.0229 (7)	−0.0153 (7)
O2	0.0431 (6)	0.0673 (9)	0.0636 (8)	0.0067 (6)	0.0181 (6)	−0.0023 (6)
O3	0.0713 (8)	0.0437 (8)	0.0575 (8)	−0.0013 (6)	0.0253 (6)	−0.0084 (6)
C1	0.0533 (9)	0.0428 (10)	0.0480 (9)	0.0091 (7)	0.0222 (7)	0.0055 (7)
C2	0.0498 (9)	0.0508 (11)	0.0437 (9)	−0.0009 (8)	0.0207 (7)	0.0027 (7)
C3	0.0446 (8)	0.0566 (11)	0.0415 (8)	0.0032 (7)	0.0184 (7)	0.0025 (7)
C4	0.0449 (8)	0.0656 (12)	0.0445 (9)	0.0057 (8)	0.0168 (7)	−0.0008 (8)
C5	0.0577 (11)	0.0790 (15)	0.0482 (10)	0.0086 (10)	0.0229 (8)	−0.0076 (9)
C6	0.0606 (11)	0.0879 (17)	0.0655 (13)	0.0192 (11)	0.0305 (10)	−0.0078 (11)
C7	0.0468 (10)	0.109 (2)	0.0684 (13)	0.0185 (11)	0.0168 (9)	−0.0101 (13)
C8	0.0478 (10)	0.0837 (16)	0.0527 (10)	0.0038 (10)	0.0144 (8)	−0.0103 (10)
C9	0.0468 (8)	0.0465 (10)	0.0464 (9)	−0.0037 (7)	0.0221 (7)	−0.0027 (7)
C10	0.0611 (10)	0.0485 (11)	0.0540 (10)	0.0013 (8)	0.0248 (8)	0.0029 (8)
C11	0.0695 (12)	0.0637 (14)	0.0588 (12)	−0.0041 (10)	0.0200 (10)	0.0138 (10)
C12	0.0808 (15)	0.0851 (17)	0.0468 (10)	−0.0192 (12)	0.0294 (10)	−0.0010 (10)
C13	0.0826 (15)	0.0838 (17)	0.0647 (13)	−0.0155 (13)	0.0449 (12)	−0.0212 (12)
C14	0.0591 (10)	0.0609 (13)	0.0611 (11)	−0.0017 (9)	0.0314 (9)	−0.0102 (9)

### Geometric parameters (Å, º)

**Table:**

S1—O2	1.4333 (13)	C6—C7	1.374 (3)
S1—O3	1.4342 (14)	C7—C8	1.364 (3)
S1—C9	1.7664 (17)	C7—H7A	0.9300
S1—C1	1.7807 (19)	C8—H8A	0.9300
F1—C6	1.349 (3)	C9—C14	1.378 (3)
O1—C2	1.210 (2)	C9—C10	1.385 (3)
C1—C2	1.528 (2)	C10—C11	1.388 (3)
C1—H1A	0.9700	C10—H10A	0.9300
C1—H1B	0.9700	C11—C12	1.374 (4)
C2—C3	1.480 (3)	C11—H11A	0.9300
C3—C4	1.391 (3)	C12—C13	1.376 (4)
C3—C8	1.399 (3)	C12—H12A	0.9300
C4—C5	1.380 (3)	C13—C14	1.391 (3)
C4—H4A	0.9300	C13—H13A	0.9300
C5—C6	1.366 (3)	C14—H14A	0.9300
C5—H5A	0.9300		
			
O2—S1—O3	118.24 (9)	C5—C6—C7	122.8 (2)
O2—S1—C9	108.90 (9)	C8—C7—C6	118.70 (19)
O3—S1—C9	108.02 (8)	C8—C7—H7A	120.7
O2—S1—C1	106.18 (9)	C6—C7—H7A	120.7
O3—S1—C1	109.15 (9)	C7—C8—C3	120.62 (19)
C9—S1—C1	105.67 (8)	C7—C8—H8A	119.7
C2—C1—S1	114.57 (12)	C3—C8—H8A	119.7
C2—C1—H1A	108.6	C14—C9—C10	122.02 (18)
S1—C1—H1A	108.6	C14—C9—S1	118.98 (15)
C2—C1—H1B	108.6	C10—C9—S1	119.00 (14)
S1—C1—H1B	108.6	C9—C10—C11	118.5 (2)
H1A—C1—H1B	107.6	C9—C10—H10A	120.7
O1—C2—C3	122.41 (16)	C11—C10—H10A	120.7
O1—C2—C1	117.92 (17)	C12—C11—C10	120.1 (2)
C3—C2—C1	119.67 (15)	C12—C11—H11A	120.0
C4—C3—C8	118.88 (18)	C10—C11—H11A	120.0
C4—C3—C2	122.52 (16)	C11—C12—C13	120.8 (2)
C8—C3—C2	118.59 (17)	C11—C12—H12A	119.6
C5—C4—C3	120.65 (17)	C13—C12—H12A	119.6
C5—C4—H4A	119.7	C12—C13—C14	120.3 (2)
C3—C4—H4A	119.7	C12—C13—H13A	119.9
C6—C5—C4	118.29 (19)	C14—C13—H13A	119.9
C6—C5—H5A	120.9	C9—C14—C13	118.3 (2)
C4—C5—H5A	120.9	C9—C14—H14A	120.8
F1—C6—C5	117.9 (2)	C13—C14—H14A	120.8
F1—C6—C7	119.2 (2)		
			
O2—S1—C1—C2	159.03 (13)	C4—C3—C8—C7	0.4 (3)
O3—S1—C1—C2	−72.49 (15)	C2—C3—C8—C7	−180.0 (2)
C9—S1—C1—C2	43.46 (15)	O2—S1—C9—C14	−23.82 (18)
S1—C1—C2—O1	−93.21 (19)	O3—S1—C9—C14	−153.41 (16)
S1—C1—C2—C3	87.07 (18)	C1—S1—C9—C14	89.87 (16)
O1—C2—C3—C4	−178.92 (19)	O2—S1—C9—C10	156.30 (15)
C1—C2—C3—C4	0.8 (3)	O3—S1—C9—C10	26.71 (18)
O1—C2—C3—C8	1.5 (3)	C1—S1—C9—C10	−90.01 (16)
C1—C2—C3—C8	−178.84 (18)	C14—C9—C10—C11	−0.6 (3)
C8—C3—C4—C5	−0.5 (3)	S1—C9—C10—C11	179.24 (15)
C2—C3—C4—C5	179.91 (18)	C9—C10—C11—C12	0.3 (3)
C3—C4—C5—C6	−0.5 (3)	C10—C11—C12—C13	0.2 (4)
C4—C5—C6—F1	−179.9 (2)	C11—C12—C13—C14	−0.4 (4)
C4—C5—C6—C7	1.7 (4)	C10—C9—C14—C13	0.4 (3)
F1—C6—C7—C8	179.8 (3)	S1—C9—C14—C13	−179.46 (16)
C5—C6—C7—C8	−1.8 (4)	C12—C13—C14—C9	0.1 (3)
C6—C7—C8—C3	0.7 (4)		

### Hydrogen-bond geometry (Å, º)

**Table:**

*D*—H···*A*	*D*—H	H···*A*	*D*···*A*	*D*—H···*A*
C1—H1*B*···O3^i^	0.97	2.23	3.183 (2)	168
C5—H5*A*···O2^ii^	0.93	2.57	3.218 (3)	127

Symmetry codes: (i) *x*, *y*+1, *z*; (ii) −*x*+1, −*y*+1, −*z*+1.
